# A Taxonomy for Three-Terminal Tandem Solar Cells

**DOI:** 10.1021/acsenergylett.0c00068

**Published:** 2020-03-23

**Authors:** Emily L. Warren, William E. McMahon, Michael Rienäcker, Kaitlyn T. VanSant, Riley C. Whitehead, Robby Peibst, Adele C. Tamboli

**Affiliations:** † National Renewable Energy Laboratory, Golden, Colorado 80401, United States; ‡ Institute for Solar Energy Research Hamelin, 31860 Emmerthal, Germany; ¶ Colorado School of Mines, Golden, Colorado 80401, United States

## Abstract

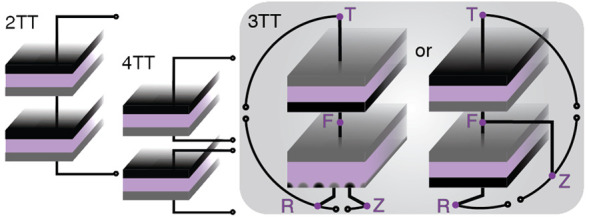

Tandem and multijunction solar cells offer the only demonstrated path to terrestrial 1-sun solar cell efficiency over 30%. Three-terminal tandem (3TT) solar cells can overcome some of the limitations of two-terminal and four-terminal tandem solar cell designs. However, the coupled nature of the cells adds a degree of complexity to the devices themselves and the ways that their performance can be measured and reported. While many different configurations of 3TT devices have been proposed, there is no standard taxonomy to discuss the device structure or loading topology. This Perspective proposes a taxonomy for 3TT solar cells to enable a common nomenclature for discussing these devices and their performance. It also provides a brief history of three-terminal devices in the literature and demonstrates that many different 3TT devices can work at efficiencies above 30% if properly designed.

Tandem or multijunction solar cells enable higher efficiencies than single-junction solar cells by absorbing different spectral ranges of sunlight more efficiently with different semiconductor band gaps to minimize thermalization losses and absorb a larger range of incident photon energies.^1,2^ Cells can be operated independently, with two terminals for each absorber, resulting in a four-terminal (4T) device for a two-absorber tandem. Cells can also be connected in series, to produce a current matched, two-terminal (2T) tandem. There has been growing interest in the concept of “three-terminal tandem” (3TT) solar cells as a way to create high efficiency and high energy yield devices. Unlike series-connected (i.e., 2T) tandems, 3TT devices do not require current matching between the subcells, enabling higher efficiency and energy yields.^3^

There are many different ways to fabricate and configure two solar absorbers with three terminals (Figure 1), and each type of tandem has different advantages and limitations in terms of manufacturing, efficiency, and energy yield. For example, “middle contact” designs require current collection from a physical contact between the two absorbers. This can avoid the need for a tunnel junction between the two materials but creates challenges with interconnections and scaling to large device areas because of the need for lateral current collection between the two absorbers. An alternative is a 3TT device with one contact on the front and two on the back, as this configuration does not require lateral current collection or electrical isolation between the layers. The most common implementation of this device is to combine a Si bottom cell with a front contact and two interdigitated back contacts (IBCs) with a 2T wide band gap top cell. However, there are many ways to create such a tandem, and there are many design trade-offs between cost, efficiency, and scalability that must be considered.

**Figure 1:**
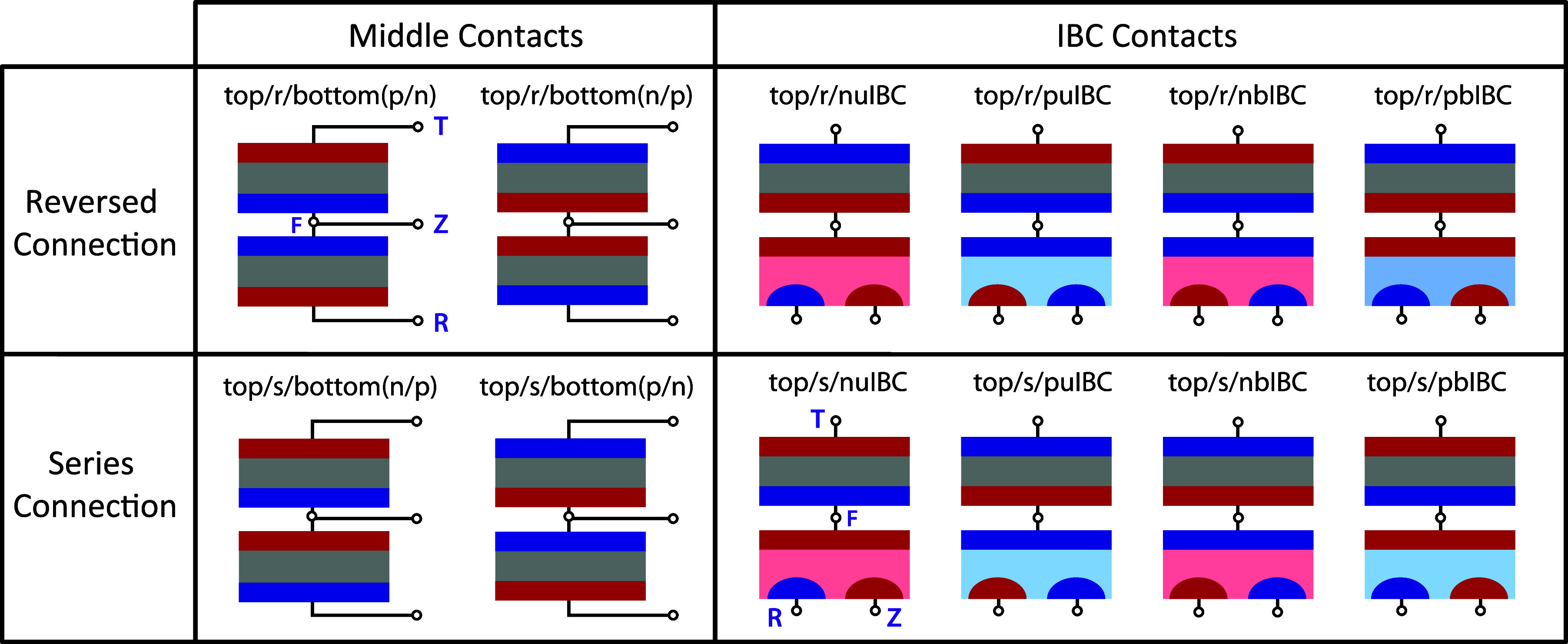
Mapping the wide variety of three-terminal tandem configurations. In all schematics, n-type materials are red and p-type materials are blue. “Top” is used as a representative top cell, and in a real device would be replaced by the name of the material, e.g. “perovskite” or “GaInP”. The naming terminology above each schematic is explained in detail in the main text. The purple letters (T, F, R, and Z) correspond to the names of the nodes used for different loading configurations.

Currently, no standard nomenclature exists to describe 3T solar cells and most studies have focused on a single device architecture, without comparing it to other configurations. Several papers have simulated or fabricated devices and show “current vs voltage” plots that do not fully define the state of the system (i.e., explicitly defining which two terminals are connected by an electrical load and the state of the third terminal during the measurement). The lack of a common nomenclature also makes it challenging to compare the design of a 3TT device where the same words are used to convey different meanings. For example, Nagashima et al. use “base” to refer to the p-type IBC contact in their cell,^4^ while Rienaecker et al. use “base” to refer to both the front and rear n-type majority carrier contact in their device.^5^ It can also be confusing because a 3T solar cell can consist of a single band gap with multiple p–n junctions^6^ or multiple single-junction absorbers interconnected in a way that results in three terminals.^7^

In this Perspective, we first propose a taxonomy that can be applied to all 3T devices to facilitate future scientific discussion. A standard naming system to describe cell components and contacts also facilitates equivalent-circuit modeling, which is needed to understand device performance and multicell interconnections. We then demonstrate how to accurately measure the cell-level performance of a 3T device, which has two different loads, adding complexity to how the performance information can be displayed. Using semiempirical simulations of device performance, we provide examples of how different load configurations and constraints can lead to different ways to visualize the performance of the same device and compare the performance for different 3TT designs. Finally, we review the history of 3T solar cell devices, discuss recent approaches that have enabled high efficiencies and robust performance under varying spectral conditions, and briefly discuss how 3TT devices can be integrated into strings and modules.

***Naming Three-Terminal Devices*.** To fully describe the performance of a three-terminal device, it is necessary to name the device and any interconnected loading circuitry. We will begin by describing the device nomenclature and then the load circuit descriptors; we then provide examples which connect the representative devices to different load circuits.

*Naming Subcell Configurations*. 3TT devices can be fabricated by combining two 2T devices with a middle contact (left column of Figure 1), or by combining a 2T top cell with a 3T bottom cell consisting of one front contact and two interdigitated back contacts (right column of Figure 1). From a taxonomy point of view, it does not matter how the electrical connection between the cells is made (e.g., wafer-bonded,^8^ mechanically bonded,^9,10^ or monolithically integrated/deposited^11^). Figure 1 shows the taxonomy for different types of 3TT devices, and this section defines the variables used to construct the terminology that is used to define the variables contained in the naming terminology above each schematic in Figure 1.

Focusing on the materials themselves, all permutations of 3TT devices can be named by considering three features of the devices:

- The relative polarity or contact carrier type of the subcells at their common interface
- The bottom cell’s polarity or absorber majority carrier type (i.e., doping)
- The number of minority carrier contacts in each subcell (equivalent to the number of p–n junctions or diodes)

For all of the 3T devices to be considered here, the top cell has only two terminals and requires only a simple descriptor, such as the placeholder name “top”, or a material (e.g., “perovskite” or “GaInP”). Although Figure 1 is limited to single-junction top cells, the top cell could also be a two-terminal series-connected multijunction cell with any number of junctions. (Theoretically the top cell could be a 3T device, but this is impractical because of increased shading from contacts and is not considered here.)

For middle contact devices (left column of Figure 1), the bottom cell is also a two-terminal device, either single-junction (as shown) or multijunction, whose polarity is indicated by standard (p/n) for “p-on-n” or (n/p) for “n-on-p” notation. The polarity of the top cell can either match the polarity of the bottom cell, or be reversed. If the polarities of the diodes in the top and bottom cells are reversed (e.g., a p/n top cell and an n/p bottom cell, where the doping sense of the connected contacts are the same) it will be described as a reversed or *r* connection (top row of Figure 1). If the top cell and bottom cell polarities match (e.g., a p/n top cell and a p/n bottom cell, where the doping sense of the connected contacts are different), a tunnel junction or metallic interconnection will be required and indicated by an *s* for “series” (bottom row of Figure 1).

By extension, single-absorber 3T-IBC cells can be integrated with a top cell in the same two ways. If the contacts of the subcells have the same type of doping at their common interface (both n/electron contacts or both p/hole contacts), it is described as a reversed or r connection. If the contacts of the subcells have the opposite dopings at their common interface (e.g., an n/electron and a p/hole contact), the cells can be connected in series and it is described as a series or s connection.

To differentiate between the many possible cell designs with IBC contacts, two additional descriptors are needed to fully describe each configuration (right column of Figure 1). The first is the majority carrier type (i.e., doping) of the bulk IBC absorber. Si solar cells are traditionally described by the doping of the wafer before processing (n-type or p-type), so the majority carrier type is indicated by n or p. (This is consistent with standard naming practices for 2T Si IBC devices.) If the absorber is intrinsic/undoped, a 3T device would be termed a 3T “iIBC.”

The second descriptor relates to the contact type for each of the three contacts in the device. Within our taxonomy, we will use “majority carrier contact” to refer to contacts that are selective for the majority carrier type in the bulk. We will use “minority carrier contact” to refer to contacts that are selective for the other carrier type. We avoid specifically naming the carrier type of the contacts (e.g., “electron contact” or “hole contact”) to enable more general comparison of 3T configurations, allowing researchers to use those terms for discussing specific cells if they so desire.

A 3T IBC subcell can have one or two minority carrier contacts (which can also be thought of as the number of diodes or p–n junctions in the device), depending on the polarity/doping of the front contact of the device. The number of minority carrier contacts is indicated *u* for “uni” for one minority carrier contact or *b* for “bi” if there are two minority carrier contacts. A uIBC cell can also be described as an IBC cell with a front surface field or front majority carrier contact. A bIBC cell can also be described as an IBC cell with a front minority carrier contact. It should be noted that because the front minority carrier contact is actively contacted in a 3TT device, this is not the same as a “floating front emitter” cell, where the front of the device is not connected to any external contact. As shown in Figure 1, these descriptors will precede “IBC” to name the possible 3T IBC permutations: nuIBC, puIBC, nbIBC, pbIBC, iIBC. Because “iIBC” provides no indication of the doping sense of the various contacts, and does not define the majority carrier type of the absorber, iIBC devices need one additional descriptor (this will be discussed below in the contact node section).

Putting this all together provides a unique and compact way to name all possible permutations of 3TT devices as shown in Figure 1. For example, the middle contact cell with a reversed connection in the upper left of Figure 1 is named “top/r/bottom(p/n),” while the IBC-contacted cell in the lower right of Figure 1 is named “top/s/pbIBC.” Without a compact taxonomy, these cells would need to be described as “a 3TT device consisting of a n-on-p top cell, and a *p*-on-n bottom cell, connected with a middle contact in an npn configuration” and “a 3TT device consisting of a top cell, connected in series with a p-type IBC cell with a conductive minority carrier contact at the front of the cell,” respectively. For concrete examples of real 3TT devices, Table S1 in the Supporting Information provides the proper taxonomy naming schemes for a subset of published 3T devices.

*The Need for Contact Node Nomenclature.* For 3T devices it is also critical to develop a consistent nomenclature for the contacts/nodes of the device to enable descriptions of the operating conditions and connections to any external circuits. While several prior 3TT solar cell publications have chosen to use the “collector,” “emitter,” and “base” convention for contact naming adapted from bipolar junction transistors (BJTs), the overlap with these names and the existing naming conventions for standard 2T solar cells becomes confusing, as was previously discussed by Cuevas and Yan.^12^ BJTs are three-terminal devices that can be used to amplify or switch a signal by controlling the current between two terminals by making small changes to the current or voltage through the third terminal.^13^ In a BJT, the emitter junction is traditionally forward biased and injects minority carriers into the base of the device. The collector junction is a reverse biased junction and collects the injected minority carriers after they pass through the base. To enable collection of all emitted minority carriers, the emitter is typically more heavily doped than the collector and base, and the base is thin to ensure carriers do not recombine.

In a solar cell, “base” is often used to refer to the lightly doped, thick region that absorbs most of the light; “emitter” is used for the heavily doped region of the opposite carrier type that forms a p–n junction, but “emitting” is not an useful description of the physics of a solar cell.^12^ The transistor-based naming convention works for some types of solar cells, but it becomes confusing for designs such as passivated contact solar cells,^14^ thin film, and III–V heterojunction solar cells.^15^ In a 3T device, each contact can collect or inject current, making the “emitter” and “collector” notation problematic.

The utility of well-defined contact nodes is that they enable compact notation to fully define the operating state of the cell. For BJTs, “common base,” “common emitter,” and “common collector” are well-defined topologies which each have different functionalities for signal amplification.^13^ To fully define the operating state of a 3T solar cell, an analogous nomenclature system is needed. Some types of 3TT devices have a majority carrier contact that is similar to the base contact in BJTs, but there are other ways of making a 3TT device that do not parallel this structure. For example, uIBC cells (with two majority carrier and one minority carrier contact) do not have the npn or pnp structure that parallels the BJT, and all the current in the cell must flow out the minority carrier contact. To avoid confusion, we propose a node naming convention for 3TT solar cells that does not overlap with that used for transistors.

*Naming the Contact Nodes*. In the 3TT taxonomy, the four possible nodes/contacts have been named: T, R, Z, and F. The node names for 3TT devices are indicated with purple text in Figures 1 and 2. Rather than use names that describe the physical properties of the device, we have chosen a naming scheme that describes the function of the node during solar cell operation. This enables qualitative discussion of devices using standard conventions without overlap or dual definitions of terms compared to BJT terminology.

**Figure 2:**
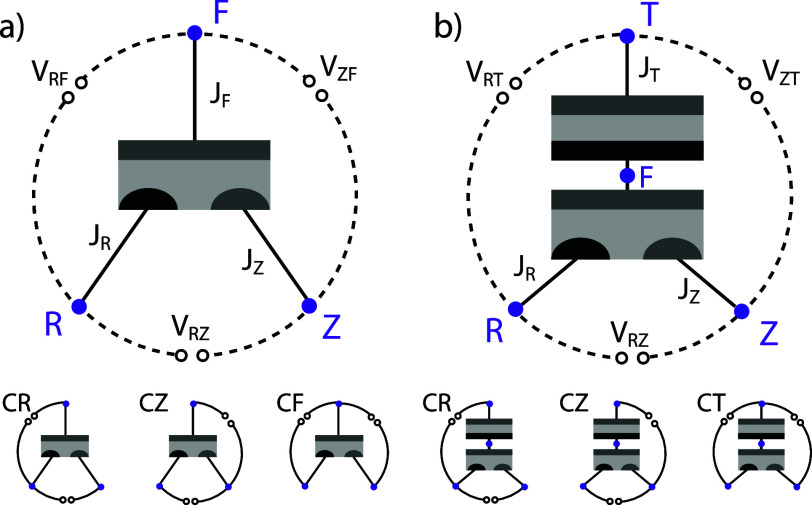
Node naming and loading conventions for (a) a standalone 3T IBC subcell and (b) a generic s-connected 3TT device. The node names are independent of the cell doping, so neutral colors are used. Each cell can be loaded in three different ways, indicated by which contact is common between the loads (CR, CZ, C(F/T)) as shown schematically below the larger diagrams. The voltage difference or current flowing between two nodes is indicated using the two subscripts of the respective nodes (e.g *V*_RT_). The current through each node is indicated with the subscript of the node (e.g., *J*_Z_).

The node/contact at the top of the top cell is T for “top” and is typically connected to an external circuit using a metal grid. The current through the top contact (*I*_T_) is therefore equal to the current generated in the top cell (*I*_top_). The F stands for “front” (of the bottom cell) or “focus” (of the tandem) and is simply a node placed between the top and bottom cells. Note that the F contact is not typically directly contacted in a tandem device but is defined for convenience and for the sake of modeling and understanding device operation.

The R stands for “root” or “raíz” (in Spanish) and is the contact of the bottom cell, through which the bottom cell current *I*_R_ = *I*_bottom_ flows. In a circuit model, R is connected to the F contact through a diode or junction. For a cell with only one back contact (e.g., any middle contact device), this is simply the contact at the back of the device. For an IBC bottom cell, the R contact will always be the contact with the doping sense that is opposite from the other two contacts (i.e., a majority carrier contact for a bIBC cell and a minority carrier contact for a uIBC cell). From an operational perspective, the bottom subcell can generate power only when the R contact is used.

The Z stands for “zusätzlich,” which means “extra” or “additional” in German, chosen because the Z contact provides a third current (*I*_Z_) to node F. For a middle contact device, Z is the middle contact, and there is always some resistance when current flows between F and Z (not shown). For an IBC device, the Z contact always has the same doping type as the front of the bottom cell (i.e., a majority carrier contact for a uIBC cell and a minority carrier contact for a bIBC cell).

A consistent node-naming scheme provides a useful way to describe the configuration of iIBC devices. If a 3T subcell has a truly intrinsic base, then one additional descriptor is needed. We have addressed this by defining the doping type of the R node, as in nR if the R contact is an electron contact or pR if the R contact is a hole contact.^12^ For example, an iIBC device with two electron contacts would be described as iIBC(pR).

*Loading Topology*. Using the T, R, Z, and F notation, the specific current and voltage being measured can always be uniquely described, eliminating confusion when cells are wired in different ways. The current or voltage being measured between two nodes can be indicated with subscripts (e.g., *V*_RZ_ = *V*_R_ – *V*_Z_ and *V*_ZR_ = *V*_Z_ – *V*_R_). It also provides an easy way to compactly define the topology of the cell (i.e., how the cell is wired to external circuits or loads).

Figure 2a shows the naming and loading options for a generic IBC-based 3T subcell, and Figure 2b shows the same contact topology for a generic 3TT device with IBC contacts. The node names do not depend on the doping of the cell, so neutral colors are used. (For an nuIBC cell, the R contact is the p-type IBC contact, while for an nbIBC cell, the R contact is the n-type IBC contact.) Dashed lines are used in Figure 2 to indicate each possible loading circuit, but having three loads on the cell simultaneously would overconstrain the system. In real operation, loads would be placed across two of the open leads, making one of the three nodes “common” (e.g., common R (CR), common Z (CZ), or common T (CT). (Note for a single-junction 3TT device, CF would replace CT.) An example of CR, CZ, and C(F/T) connections are shown below the larger current-wheel schematics for each device in Figure 2.

Just as different transistor topologies allow the same semiconductor device to be used in different ways, a 3TT solar cell can produce very different currents and voltages across loads in the different configurations discussed above. Examples of this are shown in the next section. It is critically important to understand that the operating state of a 3T device is fully determined by two independent parameters, and this can be done independently of where loads are attached to a device. For example, the power produced by the 3TT device in Figure 2b can be specified over all of its possible operating conditions as a function of two voltages (e.g., *V*_RZ_ and *V*_ZT_), or two currents (e.g., *I*_R_ and *I*_T_), or a voltage and a current (e.g., *V*_RZ_ and *I*_T_). This means that, neglecting external resistances, a cell can achieve the same maximum power point (MPP) operating in CR, CZ, or CF/T mode.

***Measuring Tandem Performance*.** The taxonomy introduced above provides an intuitive way to understand and name all the relevant currents, voltages, and loading configurations that can be measured for a 3T device. However, presenting the experimental data from such a device in a comprehensive way is often challenging. A 2T solar cell’s behavior can be fully defined with one independent variable, but a 3T device has an extra degree of freedom, requiring two independent variables. Mathematically, the current–voltage or power–voltage behavior of a 2T is described by a line, but for a 3T device, that line becomes a surface. Most researchers are accustomed to thinking about current–voltage curves for 2T devices and routinely discuss figures of merit such as open-circuit voltage, short-circuit current density, and fill factor when analyzing solar cell data. When power is generated in two separate but coupled circuits, the current–voltage data cannot be added together; therefore, the standard figures of merit are hard to define. At any operating point of the 3T device, the total power is the sum of the power simultaneously measured across two loads that are defined by the loading topology of the cell. While the currents at the two loads cannot be added, the powers can be added, enabling one output variable for two independent input variables. Therefore, one approach to fully describe the system in one graph is to plot the total power of the tandem cell as a function of the voltages (or currents) across two external loads.^16,17^

To understand the utility of plotting data in this way, we can first look at the case of a 4TT device where the top and bottom subcells can be measured independently. Figure 3a shows the current density versus voltage (*J*–*V*) and power density versus voltage (*P*–*V*) data measured for a mechanically stacked GaAs//Si 4TT device. The Si cell was measured while the GaAs cell was held at the MPP. (Note, in a real 4TT GaAs//Si tandem, the cells are optically coupled by luminescent coupling that changes with applied voltage. For simplicity, this example neglects luminescent coupling and computes the 4TT power using only this single Si-cell measurement.) Figure 3b shows the same data, but with the independent voltages for the GaAs and Si subcells plotted on different axes and the total power of the system (*P*_GaAs_ + *P*_Si_) calculated at every point.

**Figure 3:**
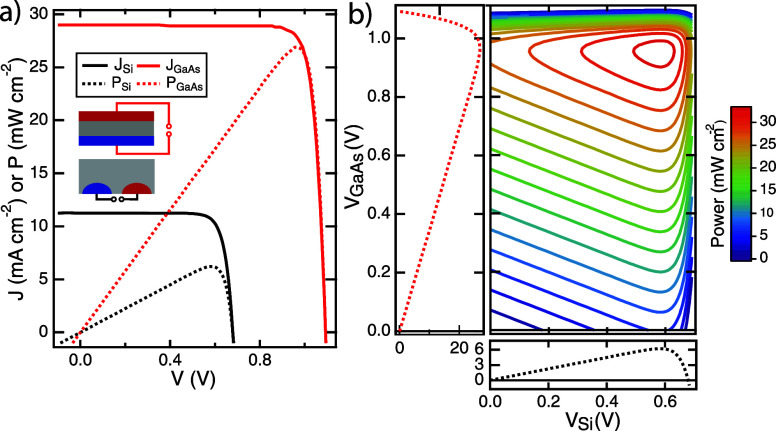
(a) Current density vs voltage and power density vs voltage plots for a 4TT GaAs//Si experimental device measured under AM1.5G illumination. (b) The same 4TT data, plotted as power contours in *V*_GaAs_ vs *V*_Si_ space.

The *P*–*V*_Si_–*V*_GaAs_ plot enables the total maximum power point of the device (in the case of the 4TT tandem in Figure 3, 32.5%) to be easily determined. If two of the terminals of a 4TT device are shorted together, then the performance can also be measured in 3T mode, as was demonstrated by Schnabel et al.^17^ (Note that in ref (17) the 4TT-as-3TT measurement was done in a mode equivalent to CR mode for a GaInP/s/nuIBC.)

The same *P*–*V*–*V* plots can be generated for any 3T device by sweeping two independent variables and calculating the total system power. For some middle-contact devices, this can be nearly identical to the 4TT (e.g., independent operation) performance if the resistance losses in contacts and wires are negligible.^18^ An IBC-based 3TT device has coupled current–voltage behavior, because the current from the top cell must be collected through one of the bottom cell contacts. Figure 4 shows the *P*–*V*–*V* and *P*–*J*–*J* data of a GaAs/s/nuIBC simulated semiempirically using experimental top cell data and TCAD simulations of the bottom 3T nuIBC cell in both CR and CZ mode (for more information on simulation details, see the Supporting Information). The load currents and voltages between two nodes are shown in Figure 4a,b for each mode. Figure 4c shows the power measured in CZ mode (*V*_ZT_ vs *V*_RZ_ axes), while Figure 4d shows the power measured in CR mode (*V*_RT_ vs *V*_RZ_ axes). Panels e and f of Figure 4 show the same data plotted as a function of the current densities across each load. These load currents can be related to the device currents (in Figure 2) by current conservations but this relationship will be different for CZ, CR, and CT modes. The maximum power generated by these devices is the same in the two modes, but the operating state of the loads is very different. In CZ mode, both circuits produce power simultaneously at the global MPP. However, in CR mode, power is being injected into the RZ circuit to enable the system to achieve its overall maximum power. This type of injection behavior for CR loading can occur for any tandem configuration where the bottom cell is current-limiting.

**Figure 4:**
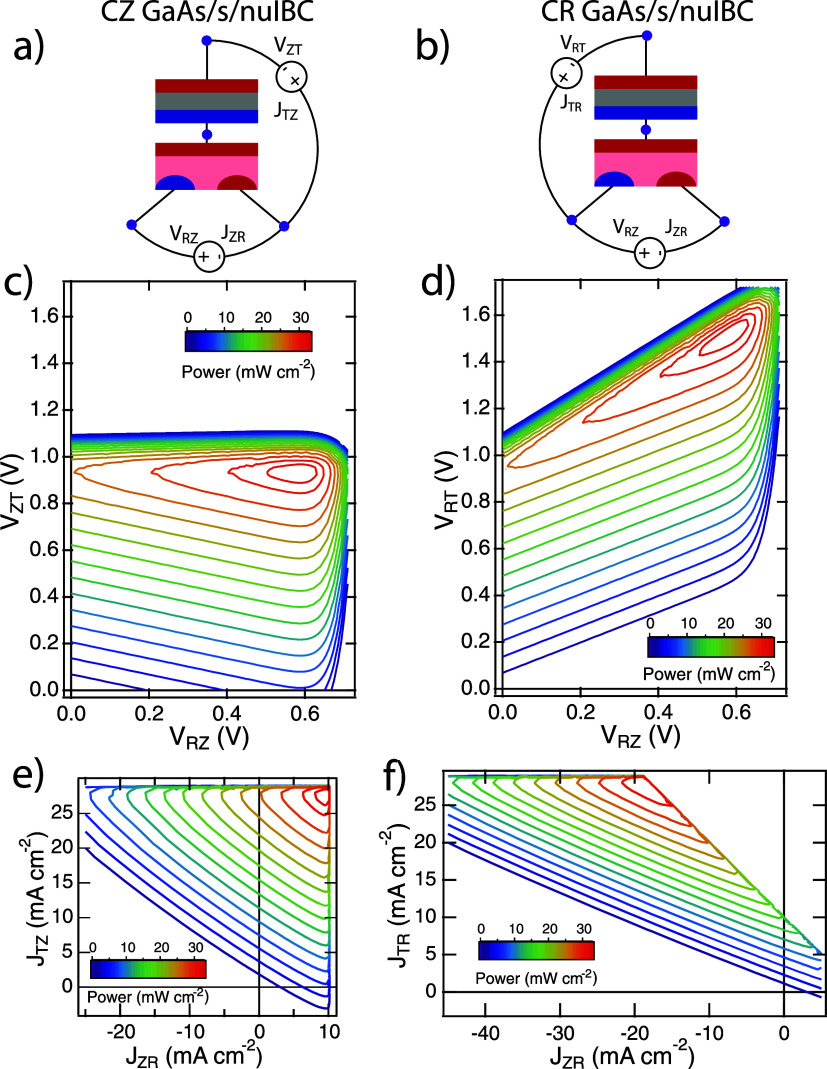
Simulated 3TT GaAs/s/nuIBC-Si device power contours under AM1.5G illumination. (a) Schematic of CZ loading; (b) schematic of CR loading; (c) CZ *P*–*V*–*V* plot; (d) CR *P*–*V*–*V* plot; (e) CZ *P*–*J*–*J* plot; (f) CR *P*–*J*–*J* plot. In the CZ case, both loads are producing power at the max power point, but in the CR case, the load across the R and Z nodes is injecting power into the device to achieve the same operating state (hence negative currents for *J*_ZR_). (This simulation neglects luminescent coupling between the cells.)

***A Brief History of 3T Devices*.**To enable deeper understanding of 3TT devices, it is important to put the prior approaches to 3TT devices in context with a uniform naming convention. Many of the more recent device designs are topologically identical to work proposed decades earlier. To advance the field, it is important to understand prior work in a context where different designs can be directly compared. Table S1 of the Supporting Information tabulates the naming conventions for the previously published devices mentioned below in chronological order.

*Middle Contact 3T Devices*. The first generation of proposed 3TT devices consisted of double-junction (i.e “two color” or “wavelength division”) III–V solar cells where a middle contact was used as a common ground to extract current from two subcells.^7,19−26^ This concept has also been proposed on polymer solar cells and hybrid II–VI/III–Vs tandems,^27,28^ as well as multijunction stacks.^29^ These devices had ((p/n)–(n/p)) or ((n/p)–(p/n)) structures so did not require the growth of tunnel junctions, making them r-connected devices operated in the CZ configuration. Middle contact cells including a tunnel junction (i.e., s-connected) have also been proposed and fabricated where a third contact is added between the cells. In this configuration, a middle contact can be used to characterize the behavior of individual subcells and the tunnel junction within a 2T multijunction stack.^30−32^

While most middle contact approaches have relied on a heavily doped, majority carrier lateral conduction layer between the cells, Marti and Luque proposed a 3T “hetero-junction bipolar transistor” solar cell where a base material is a common contact between two different heterojunction diodes.^33^ An experimental demonstration of this device with the transistor effect minimized is equivalent to an r-connected middle contact 3TT device.^34,35^

In practice, most experimentally demonstrated 3TT cells with a middle contact have been made using two contacts at the front of the device, which limits efficiency because of shading losses and areal mismatch between the subcells. For some materials (e.g., III–Vs), the entire device stack can be monolithically grown and then processed to isolate the middle contact. It is also possible to mechanically combine cells that are grown separately.^32^

*IBC 3T Devices*. 3T devices with interdigitated back contacts have been proposed, simulated, and/or demonstrated by a variety of groups. In this configuration, the bottom cell (usually Si) has three terminals and does not have simple current–voltage behavior that is typical of a 2T solar cell, as the current–voltage behavior of the three contacts are coupled.^5,16,36^ However, most studies in the literature represent the performance of the devices with standard *J–V* plots. Some provide information about the current or voltage at the third contact, but few demonstrate that the plots they are showing are actually representative of the maximum power of the device. 3T IBC devices have also been used in electrochemical systems to probe fundamental material properties or combine photoelectrochemical processes with electricity generation.^37,38^

Most studies have focused on combining uIBC bottom cells (with a majority carrier contact at the front of the bottom cell) with wide band gap III–V or perovskite material as a way to get around the need for tunnel junctions and/or current matching.^4,39−41^ The widely cited work by Nagashima et al. was based on top-cell/r/puIBC devices, where the top cell was either AlGaAs(n/p) or a 2J tandem of GaInP/GaAs(n/p), and the bottom cell was based on either Si or Ge.^4^ More recently, our group has demonstrated a GaInP/s/nuIBC cell where the cells are fabricated separately and combined with a transparent conductive adhesive (TCA),^10^ resulting in an efficiency of 27.3%,^42^ and others have taken similar approaches with GaAs/s/nuIBC cells.^43^

There have been fewer studies focusing on 3TT devices with a bIBC bottom cell. The concept of a bIBC 3T Si cell was first proposed in 1978, without a higher band gap top-cell, by exploiting the transistor effect.^6^ Recent modeling and experimental work has shown that it is possible to operate a bIBC 3T Si device with very little loss.^36,44^ However, there have been prior reports that incorrectly assume that the power in the RZ (i.e., IBC) circuit of a perovskite/s/pbIBC cell can be extracted independently of the state of the top cell, which greatly oversimplifies the operation of this device.^45^ Because a pbIBC Si subcell may be less expensive to fabricate than an nuIBC subcell, from a manufacturing perspective, it is important to compare the behavior of uIBC and bIBC cells.^44^ Simulations comparing the performance of 3TT devices fabricated from these two types of IBC subcells are presented in the next section.

***Future Prospects for IBC-Based 3TT Devices*.** Given the wide variety of ways in which IBC-based 3TT performance data has been presented in the literature, we present here TCAD simulations of a variety of 3TT configurations in an effort to directly compare them using a standardized measurement format. IBC-based 3TT devices have an advantage over 2T tandems in that the cells do not need to be current-matched. IBC-based 3TT devices have the potential to have higher efficiency and energy yield than 4TTs because the top and bottom cells can be interconnected with less shading and resistive losses when current does not need to be laterally extracted between the subcells.^46^ While there will clearly be challenges with designing and fabricating new types of tandems, the important point is that all types of tandems should function at similar maximum efficiencies for a “well-designed” device.

Figure 5 shows *P*–*V*–*V* contours for four types of simulated 3TT devices combining a GaInP top cell and a Si bottom cell, which are examples of well-behaved solar cell materials; the general trends will also apply to a broader set of subcells. All data are plotted in CZ mode, which facilitates understanding because both loads in this topology generate power. Figure 5a shows the GaInP/s/nuIBC cell that has been previously published by our group.^3^ If the polarity of the top cell is reversed, the device becomes a GaInP/r/nuIBC cell (Figure 5b). If the bottom cell is changed to a pbIBC cell (i.e., changing the bulk doping from n to p), the resulting tandem is a GaInP/s/pbIBC (Figure 5c) or a GaInP/r/pbIBC (Figure 5d), based on the type of connection at the F node. All the data have been plotted with the maximum power production in the first quadrant. More details about the simulations of these devices and the equations to calculate the device performance for each configuration can be found in the Supporting Information. All of these configurations produce a tandem device efficiency over 31% under AM1.5G 1-sun conditions (see Table 1).

**Figure 5:**
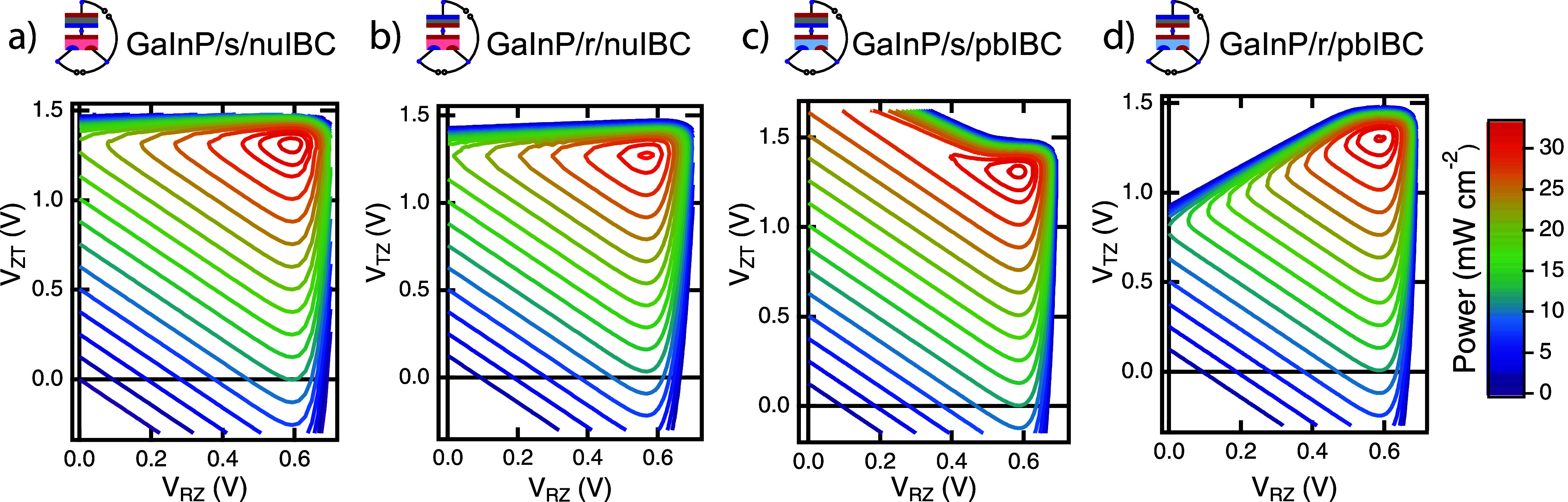
Simulated CZ *P*–*V*–*V* contours for multiple configurations of a 3TT device based on GaInP and Si (under AM1.5G illumination): (a) GaInP/s/nuIBC, (b) GaInP/r/nuIBC, (c) GaInP/s/pbIBC, and (d) GaInP/r/pbIBC.

**Table 1:** Voltages at MPP Across the Loads for GaInP/IBC-Si Tandem 3TT Devices in CZ and CR Modes^a^

		CZ mode		CR mode	
cell configuration	η_3TT,max_ (%)	*V*_RZ_ (mV)	*V*_ZT/TZ_ (mV)	*V*_RZ_ (mV)	*V*_RT/TR_ (mV)
GaInP/s/nuIBC	32.3	591	1309	591	1900
GaInP/r/nuIBC	31.5	568	1272	568	704
GaInP/s/pbIBC	32.2	580	1311	580	1891
GaInP/r/pbIBC	32.1	580	1303	580	723

a Voltages are reported as positive, although the current flows in opposite directions for s- and r-connected 3TT devices.

GaInP is an almost current-matched top cell for Si and is current-limiting in the data presented above, but many candidate top cell materials have narrower band gaps. It is important to look at 3TT devices where the bottom cell is current-limiting to understand applicability to narrower band gap cells in general. Figure 6 shows the same tandem configurations as Figure 5a,d, but with a GaAs top cell. GaAs has a narrower band gap than GaInP and results in a tandem where the bottom Si cell is current limiting. As discussed above, this means that in the CR mode of operation, the RZ load must inject power into the device to enable its overall maximum power point. In CZ mode, both loads produce power at the maximum power point. The CZ *P*–*V*–*V* plot for GaAs/r/nuIBC is shown in Figure 6a, and that for GaAs/r/pbIBC is shown in Figure 6b. Note the overall efficiency is nearly the same as for the GaInP device (over 31% under 1-sun AM1.5G conditions).

**Figure 6:**
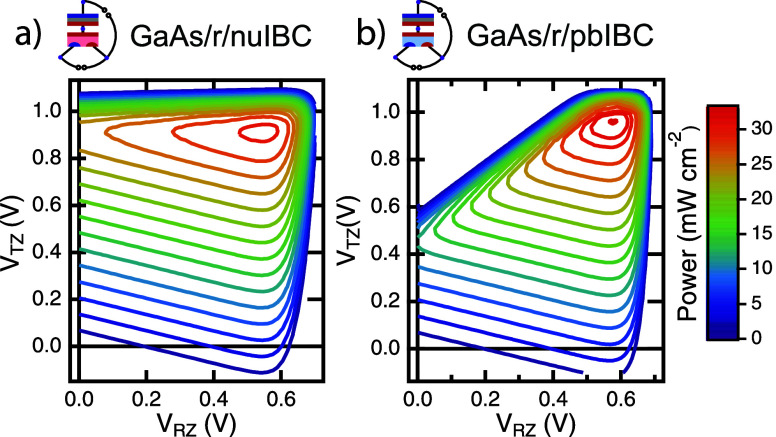
Simulated CZ *P*–*V*–*V* contours for a subset of configurations of a 3TT device based on GaAs and Si (under AM1.5G illumination): (a) GaAs/r/nuIBC and (b) GaAs/r/pbIBC.

All configurations of 3TT devices, with different bottom cells, top cells, and interconnections between the cells are capable of achieving over 31% efficiency in these simulations. However, the currents and voltages measured across the loads are different for each configuration, as can be seen from the different shapes of the power contours in Figures 5 and 6. To better quantify this, Table 1 shows the voltages measured across each load at MPP in both CZ and CR configurations for 3TT devices composed of GaInP and 3T Si subcells. For the same top and bottom subcell pair, we see slightly lower overall efficiency for the r connections than in the s-connected case, because the IBC bottom cell has more current flowing through its Z contact at MPP in the r-connected case, leading to higher resistive losses. This is expected based on prior simulations, but it is important to consider the relative performance between different types of cell configurations.^16,36^ The numerous ways to report a measurement of the MPP of a 3TT device reinforce the need for a standard taxonomy, and also have implications for how multiple 3T tandems can be interconnected into strings.

*Interconnections and Energy Yield*. Unlike 2T and 4T tandems, a 3T device does not have an easily replicated unit cell that can be connected together in series. This makes interconnections of individual cells into strings and energy yield modeling at the string level more difficult than for the 2T or 4T case. It is possible to connect 3TT cells into strings using voltage-matching and/or complementary cell approaches, and several topologies have been proposed by different groups.^47,48^

A wide variety of energy yield studies for tandems have been published, taking different variables into account.^49−51^ While summarizing this work is beyond the scope of this Perspective, it should be noted that all studies have used equivalent-circuit models to define the cell performance. No energy yield modeling has taken the unique properties of IBC-based 3TT devices into account, instead using standard single-diode or two-diode models to represent the solar cell performance.

On the basis of band gap alone, 4TTs have the best projected efficiencies and energy yield,^52^ but actual 4T tandems will have losses due to shading and/or resistance loss due to the need for TCOs or metal grids between the subcells. The resulting loss in energy yield can range from 5 to 15% depending on the configuration of the grids and will increase with larger cell sizes.^46^ 4TTs can be connected as two independent strings—requiring two different inverters—or combined through voltage-matching approaches.^53,54^

Several approaches have been proposed for how to integrate “middle-contact” 3TT devices,^47^ but there has been less work on experimentally verifying this approach.^55^ It is known that voltage-matched (2T at the module level) approaches with 3T devices will require some finite losses at the end of the strings, but most energy yield models to date have focused on infinitely long strings, without explicitly quantifying the losses of the device.^48^ There are several known ways to string 3T devices together, but realistic models will need to take specific cell-level device outputs into consideration to appropriately design 3T strings and modules. Understanding how the type of tandem, the contacting approach, and the interconnection scheme impact the potential energy yield of a device is the next important step for energy yield modeling of 3T PV devices.

*Summary*. 3TT devices have a long history, but only recently have there been experimental demonstrations of these devices and studies focusing on energy yield of different tandem configurations. Numerous reports show that specific subcell combinations can produce 3TT efficiencies that are comparable or better than those of 4TT devices (once shading losses are accounted for). There will, however, always be a trade-off between cell efficiency, module efficiency, and balance of system costs that must be considered for each 3TT configuration. It remains to be seen whether the energy yield advantages of a 3TT or 4TT device lead to lower levelized cost of electricity production than a 2T device that can be made into a string or module with less integration-related losses.

We have proposed a taxonomy for 3TT devices that enables different design approaches to be compared and discussed, and we provide a condensed syntax to facilitate equivalent-circuit and energy-yield modeling. While there has been a recent flurry of interest in 3TT architectures, many approaches have oversimplified the performance or under-specified the system when reporting efficiency measurements. We hope that researchers interested in the future of high-efficiency three-terminal photovoltaics will adopt this taxonomy in their future publications to facilitate improved communication about this type of device.

## Supporting Information Available

Examples of our taxonomy applied to 3TT devices reported in the literature, simulation details, and the sign conventions and equations used to calculate the power through each load for the example devices discussed in the text (PDF)
