# Supplementary material for: A Taxonomy for Three-Terminal Tandem Solar Cells
Source: ACS Energy Lett. 2020 Mar 23;5(4):1233–42. doi: 10.1021/acsenergylett.0c00068 (PMC10906945; doi:10.1021/acsenergylett.0c00068)
Supplement: Supplementary file 1 — nz0c00068_si_001.pdf [file nz0c00068_si_001.pdf]

Supporting Information: A taxonomy for three terminal tandem solar cells

Emily L. Warren,*,† William E. McMahon,† Michael Rienäcker,‡ Kaitlyn T. VanSant,†,¶ Riley C. Whitehead,† Robby Peibst,‡ and Adele C. Tamboli†

†*National Renewable Energy Laboratory*

‡*Institute for Solar Energy Research Hamelin*

¶*Colorado School of Mines*

E-mail: emily.warren@nrel.gov

Supporting Information Available

3TT devices from the literature

Many different variations of 3TT devices have been reported in the literature. While the following list in Table S1 is not exhaustive, it provides a summary of how different proposed devices would be named using our 3TT taxonomy. Papers are listed in chronological order by first author. The reference number from the main text, device taxonomy, and indication of whether report was based on simulated or experimental results are provided for each publication.

Simulation Details

All of the simulated Si data presented was generated using the methods described by Warren et al. in prior publications.[1,2] The simulated performance of these device closely

Table S1: Summary of literature reports of 3T and 3TT devices listed in chronological order. The taxonomy for each device is listed, as well as whether the paper produced an experimental device or was a simulation.

Author/Year	Ref.	Experiment or Simulation	3TT Taxonomy
Chiang 1978	6	Experiment	Single junction pbIBC Si
Sakai 1980	19	Simulation	InP/r/InGaAsP(p/n)
Flores 1983	20	Experiment	AlGaAs/r/GaAs(n/p)
Fraas 1984	22	Patent	GaAsP/r/GaAsSb(p/n)
Wanlass 1991	24	Simulation	InP/r/GaInAs(p/n)
Soga 1996	25	Experiment	GaAs/r/Si(n/p)
Nagashima 2000	4	Simulation	AlGaAs/r/puIBC-Si
Sista 2010	27	Experiment	P3HT/r/PSBTBT(p/n)<br>P3HT/r/PSBTBT(n/p)
Emziane 2011	29	Simulation	GaAs/r/Ge(p/n)<br>GaAs/r/Ge(n/p)<br>AlGaAs/r/Si(p/n)
Alshkeili 2014	28	Simulation	CdTe/r/InGaAs(p/n)
Marti 2015	33, 34	Simulation	generic top/r/bottom<br>(both n/pand p/n)
Adhyaksa 2017	45	Simulation	perovskite/r/pbIBC
Warren 2018	3, 16	Simulation	GaInP/s/nuIBC
Soeriyada 2018	31	Experiment/ Simulation	GaAsP/s/SiGe(n/p)
Zou 2018	39	Simulation	GaNP/r/nuIBC-Si
Rienacker 2019	5	Experiment/ Simulation	Single junction nuIBC Si
Zhang 2019	35	Simulation	generic top/r/bottom (n/p)
Santbergen 2019	40	Simulation	perovskite/r/nuIBC<br>perovskite/s/nuIBC
Djebbour 2019	41	Simulation	GaInP/r/nuIBC
Park 2019	32	Experiment	perovskite/s/Si(n/p)
Stradins 2019	36	Simulation	generic nuIBC, pbIBC
Rienaecker 2019	44	Experiment	single juncsion pbIBC
Schnabel 2019	42	Experiment	GaInP/s/nuIBC
Tayagaki 2019	43	Experiment	GaAs/s/nuIBC

matches experimental measurements of 3T POLO devices fabricated at ISFH[3,4] and 3TT GaInP/t/nuIBC devices fabricated at NREL.[5] This framework can be used to directly compare different top-cell materials that could be combined with 3T Si bottom cells.

Briefly, the simulation process is outlined in Figure S1. Optical simulations of the generation profile within the Si sub-cell under different top-cells were carried out using SunSolve ray-tracing software from PVLighthouse.[6] All optical data used in these simulations is publicly available or experimentally measured for each material in the stack. This generation profile was then used within a TCAD Sentaurus (Synopsis) simulation package run in mixed mode to calculate the performance state of the the Si device. This approach does not account for luminescent coupling between the cells, but we find that effect is only significant when the top-cell is operated at very low currents, and that does not impact the global maximum power point of the tandem, when both sub-cells are operated near their maximum power points. The tandem device performance was then calculated semi-empirically by combining NREL-certified experimental current-voltage data of the relevant top cell and calculating all states of the system where the currents through the F node of the device are equal. Simulated current data is scaled based on the area of the simulated devices (I_{sim}/area$_{3TT}$), and therefore reported as current density (J [mA/cm^2]) throughout the main text.

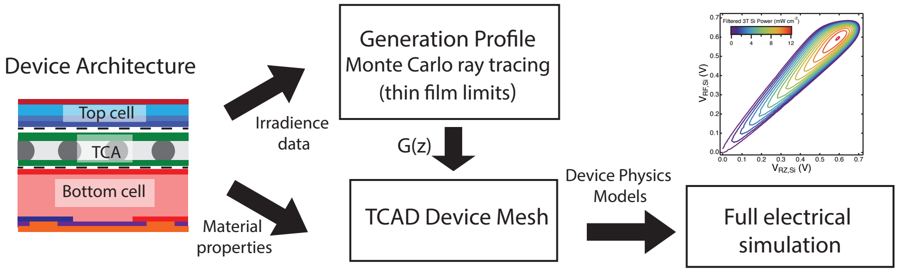

Figure S1: Schematic of process flow for generating simulated performance data.

The sign convention for calculating power through each load was set so that the maximum power point of the cell would always graphically be in the first quadrant to allow direct comparison between configurations. The equations below apply to nuIBC- or pbIBC-based tandems. The signs would be reversed for puIBC- or nbIBC-based tandems. For s

connections, $V_{topcell}$ = (V_F - V_T). For *r* connections, $V_{topcell}$ = (V_T - V_F).

For *s* connection *CR* mode (Eq. 1):

$$P_{RZ} = J_Z * (V_R - V_Z);\ P_{RT} = J_{topcell} * (V_{topcell} + V_R - V_F) \tag{1}$$

For *s* connection *CZ* mode (Eq. 2):

$$P_{RZ} = J_R * (V_R - V_Z);\ P_{ZT} = J_{topcell} * (V_{topcell} + V_Z - V_F) \tag{2}$$

For *r* connection *CR* mode (Eq. 3):

$$P_{RZ} = J_Z * (V_R - V_Z);\ P_{TR} = J_{topcell} * (V_{topcell} + V_F - V_R) \tag{3}$$

For *r* connection *CZ* mode (Eq. 4):

$$P_{RZ} = J_R * (V_R - V_Z);\ P_{TZ} = J_{topcell} * (V_{topcell} + V_F - V_Z) \tag{4}$$

References

(1) Warren, E. L.; Deceglie, M. G.; Rienäcker, M.; Peibst, R.; Tamboli, A. C.; Stradins, P. Maximizing tandem solar cell power extraction using a three-terminal design. *Sustainable Energy & Fuels* **2018**, *2*, 1141–1147.

(2) Warren, E.; Rienäcker, M.; Schnabel, M.; Deceglie, M.; Peibst, R.; Tamboli, A.; Stradins, P. Operating principles of three-terminal solar cells. *Proceedings of the 46th IEEE WCPEC* **2018**, 2648–2650.

(3) Rienäcker, M.; Warren, E. L.; Schnabel, M.; Schulte-Huxel, H.; Niepelt, R.; Brendel, R.; Stradins, P.; Tamboli, A. C.; Peibst, R. Back-contacted bottom cells with three terminals: Maximizing power extraction from current-mismatched tandem cells. *Prog. Photovoltaics* **2019**, *27*, 410–423.

(4) Rienäcker, M.; Warren, E. L.; Wietler, T. F.; Stradins, P.; Tamboli, A. C.; Peibst, R. Three-Terminal Bipolar Junction Bottom Cell as Simple as PERC: Towards Lean Tandem Cell Processing. *Proceedings of 46th IEEE PVSC* **2019**,

(5) Schnabel, M.; Schulte-Huxel, H.; Rienäcker, M.; Warren, E. L.; Ndionne, P.; Nemeth, B.; Klein, T. R.; van Hest, M. F. A. M.; Geisz, J. F.; Peibst, R.; Stradins, P.; Tamboli, A. C. Three-terminal III–V/Si tandem solar cells enabled by a transparent conductive adhesive. *Sustainable Energy & Fuels* **2019**,

(6) PVLighthouse and SunSolve™, *https://www.pvlighthouse.com.au*; accessed 25 November 2019.
